# Effect of Excessive Body Weight and Emotional Disorders on the Course of Pregnancy and Well-Being of a Newborn before and during COVID-19 Pandemic

**DOI:** 10.3390/jcm10040656

**Published:** 2021-02-09

**Authors:** Artur Wdowiak, Marta Makara-Studzińska, Dorota Raczkiewicz, Paula Janczyk, Aneta Słabuszewska-Jóźwiak, Anita Wdowiak-Filip, Noemi Studzińska

**Affiliations:** 1 Diagnostic Techniques Unit, Medical University of Lublin, ul. Staszica 4/6, 20-081 Lublin, Poland; wdowiakartur@gmail.com; 2 Department of Health Psychology, Jagiellonian University Medical College, ul. Kopernika 25, 31-501 Kraków, Poland; marta.makara-studzinska@uj.edu.pl; 3 Department of Medical Statistics, Center of Postgraduate Medical Education, School of Public Health, Kleczewska 61/63, 01-826 Warsaw, Poland; dorota.bartosinska@gmail.com; 4 Nursing and Midwifery Institute, Jagiellonian University Medical College, Kopernika 25, 31-501 Kraków, Poland; paula.janczyk@uj.edu.pl; 5 First Department of Obstetrics and Gynecology, Center of Postgraduate Medical Education, Żelazna 90, 01-813 Warsaw, Poland; 6 Department of Dermatology, Venerology and Pediatric Dermatology, Medical University of Lublin, Radziwiłłowska 13, 20-080 Lublin, Poland; anita.wdowiak@gmail.com; 7 Green Medica, Magiczna 6, 03-289 Warsaw, Poland; emi.studzinska@gmail.com

**Keywords:** obesity, BMI, pregnancy outcome for mother and child, depression, COVID-19 pandemic

## Abstract

This study aimed to evaluate whether excessive body weight and the COVID-19 pandemic affect depression, and subsequently whether depression, excessive body weight, and the COVID-19 pandemic affect the course of pregnancy, as well as the well-being of a newborn. The research material included data retrieved from the medical records of 280 pregnant women who were provided with care by medical facilities in Lublin (100 women with normal weight, 100 overweight women, 50 with Class I and 30 with Class II obesity). They completed a Beck depression inventory (BDI) in pregnancy twice, in order to assess the risk of occurrence of postpartum depression. Pre-pregnancy BMI positively correlated with the severity of depression, both at 10–13 weeks of pregnancy (*p* < 0.001), and at 32 weeks of pregnancy (*p* < 0.001). The higher the pre-pregnancy BMI, on average the higher the severity of depression. The severity of depression was significantly higher during the pandemic than before it in women with normal body weight before pregnancy (*p* < 0.001), as well as in those overweight (*p* < 0.001) and with Class II obesity (*p* = 0.015). Excessive body weight before pregnancy leads to depressive disorders during pregnancy, increases the risk of preterm delivery, and exerts a negative effect on the state of a newborn. Depressive symptoms among pregnant, overweight and obese women intensified during the COVID-19 pandemic.

## 1. Introduction

The state of health of a newborn after birth depends, to a great extent, on the course of pregnancy. The course of pregnancy is conditioned, one the one hand, by the health problems of the mother, and on the other hand, by proper care provided by medical staff. Some health problems, such as an excessive body weight, imply the occurrence of complications during pregnancy, and may pose a threat for health and life of a newborn [1,2,3].

Pregnancies in women with excessive body weight are considered as high risk pregnancies. In obese women pregnancy brings about risk in the form of arterial hypertension, gestational diabetes, urinary and reproductive tract infections, pre-eclampsia, and eclampsia, which cause certain risks for the fetus and newborn, such as: miscarriage, preterm labor and premature birth, intrauterine infection, too high birth weight, and the possibility of perinatal injury [2].

Many studies have shown that obesity frequently co-occurs in patients with depressive disorders. It was also observed that the relationship between BMI and mood disorders is especially strong in obese patients and in women. The results of the study, Global Burden of Disease 2010, suggest that both obesity and mental disorders belong to the most important contemporary health problems, and their co-occurrence may pose a serious threat for pregnant women and their children [4].

Many scientific studies from the last few years have confirmed the interrelation between depression and obesity during pregnancy. The risk of depression is higher in overweight women, and is especially elevated among obese women with BMI ≥30 [5,6,7,8,9,10]. In turn, Insan et al. [11], in their study conducted among women of British origin and Asian women, indicated a lack of any relationship between pre-pregnancy BMI and depression, similarly to the study by Molyneux et al. [12] on women from Australia, Ireland, New Zealand, and the UK. Despite a large number of available studies, emerging inconsistencies suggest that further research is important, especially with consideration of the same scales for the assessment of depression, and factors strongly mediating the intensity of depressive symptoms.

Pregnancy is not a protective factor against mood disorders, therefore, depression in pregnancy was often unnoticed and too rarely diagnosed [12]. Attention was focused primarily on the postpartum period, which was associated with a relatively large number of studies. Considerably less attention was devoted to depressive disorders [13,14].

However, the results of studies confirm that during pregnancy there often occurs an exacerbation of the course of existing psychological disorders, and the development of new cases [15,16]. The occurrence of a psychological disorder during pregnancy and the postpartum period may have important long-term consequences, not only for the woman and her child, but also for the whole family.

Research from the last 20 years has indicated a possible, although still equivocal [15], relationship between psychological distress in the mother during the perinatal period and complications occurring in the newborn. In a review of studies Alder et al. emphasized that depression during pregnancy is an independent risk factor for concomitant obstetric, fetal, and neonatal complications [17]. The latest studies demonstrate that untreated depression in pregnancy may be associated with worse prenatal care, obstetric complications, the danger of taking medications in an uncontrolled manner, overuse of psychoactive substances, disturbed mother–child relationship, and an increased risk of the recurrence of depression during the postpartum period [18,19]. Maternal health, nutrition, and stress are linked to the subsequent development of cardiovascular disease, stroke, and diabetes in prenatally exposed offspring [20,21,22]. The associations between maternal mood in pregnancy and altered child outcome were illustrated in studies which included war experience [23], famine [24,25], and acute disasters, such as an earthquake [26], the Chernobyl nuclear disaster [27], and World Trade Center attack [28]. A long-term intrauterine exposure to glucocorticosteroids, including cortisol, leads to fetal programming changes [29] and, in consequence, to the development of cardiometabolic diseases [30] or obesity in adult life [31].

In addition, it turns out that anxiety and depression may exert an inhibitory effect on the activity of the cerebral cortex, which may potentially lead to a decreased oxytocin secretion via the hypothalamic–pituitary–adrenocortical axis, leading to weak uterine contractions. Apart from this, anxiety may reduce the secretion of norepinephrine, which may potentially lead to enhanced sensitivity to pain in pregnant women. As a result of these two mechanisms there may occur difficulties during labor, hypoxia in the newborn, as well as an increased risk of neonatal asphyxia and postpartum bleeding [32].

Women experience affective disorders twice as frequently as men, and the peak incidence is in the reproductive age, between 25–44 years [33,34]. As mentioned above, older studies confirmed that pregnancy is a protective period for depression; however, newer studies, including those that were prospective [35], indicated that the percentage of depressions is close to that in the general population of women at this age (approximately 10%), both with respect to mild and severe depressive episodes [36].

In the studies conducted to-date the frequency of occurrence of depressive symptoms in pregnancy was from 7 to even 20% [37,38,39], which depended to a large extent on the methodology and diagnostic instruments applied. In a literature review from 2004, carried out by Benett et al., the mean frequency of depression was 7.4% in the first, 12.8% in the second, and 12.0% in the third trimester of pregnancy, respectively [38]. Nevertheless, it should be noted that the majority of studies conducted to-date have referred to only one moment of pregnancy. Generally, the studies carried out to-date indicate the importance of psychosocial factors in the development of perinatal mood disorders; however, it should be emphasized that the majority of the studies considered the postpartum period, or only one of the trimesters of pregnancy; primarily the third trimester [40,41].

The state of pandemic caused by spread of the SARS-CoV-2 virus is a situation posing a serious challenge for the health care systems of countries around the world [42]. The risk factors of high levels of stress, anxiety, and confusion are, among other things, limiting the availability of health care facilities, and causing a decline in the efficiency of regional health and epidemiologic care systems. The COVID-19 pandemic may be associated with the occurrence of psychological symptoms in pregnant patients, such as: anxiety, depressive symptoms, or post-traumatic stress disorder [43,44,45,46,47,48,49]. This is related to the functioning of the human psyche. The SARS-CoV-2 virus is an extraordinary factor, a biological stressor of supra-regional character, confronting everyone with death, and against which the protective mechanisms used so far have failed; the global character of the stressor brings about consequences for human existence worldwide, and goes beyond the image of a natural or man-made disaster studied to date. The psychological and psychiatric consequences of the pandemic, with unprecedented acceleration of transmission of the virus throughout the whole world as a result of globalization, climatic changes, and the speed of population mobility, evoke a dominant, subjectively experienced, actual sense of being threatened by others, as well as fear, uncertainty, and anxiety. These symptoms lead to mood disorders on the spectrum of depressive disorder, and resulting from poor general medical status associated with treatment difficulties, due to the pandemic. In the last year several scientific reports concerning the unfavorable effect of the COVID-19 pandemic on the health of pregnant women through intensification of the symptoms of depression have appeared [43,44,45,46,47,48,49]. However, the researchers did not analyze how this situation affects the state of a newborn and the course of pregnancy.

The study aimed to evaluate whether excessive body weight and the COVID-19 pandemic affect depression, and subsequently whether depression, excessive body weight, and the COVID-19 pandemic affect the course of pregnancy, as well as the well-being of a newborn.

## 2. Materials and Methods

### 2.1. Study Group

The organizers of the study turned to eight managers of outpatient departments in Lublin who provide care for pregnant women, requesting their consent for the study. Acceptance was obtained from four medical facilities. A meeting was subsequently organized with physicians taking care of pregnant women in order to give instruction about the data which should be collected for the study and the method of recruitment of women. The recruitment consisted in placing an invitation on the websites, as well as at the reception, of medical facilities. Participation in the study was anonymous, voluntary, free of charge, and enabled resignation at any time. The women signed an informed consent to participate in research. Information was collected by physicians who provided care for pregnant women, the data were coded and passed to the investigators in an anonymous form.

Pre-pregnancy BMI was obtained based on an interview carried out by a physician, then compared with the BMI determined at the first visit in weeks 4–6 of pregnancy. If the discrepancy between body weights declared by the patient as pre-pregnancy BMI was higher than 1 kg, compared to the weight at the first visit, the data was not analyzed. Data on postpartum complications and the state of newborns, were collected by doctors providing prenatal care.

A total of 458 women agreed to participate in the study, while 178 women were excluded (32 due to the discrepancies between pre-pregnancy BMI and the BMI at the beginning of pregnancy, 54 because to the established criteria of exclusion before qualifying for the study, 54 after qualification, and 38 due to incompleteness of data).

Finally, 280 medical histories were analyzed in the study (100 women with normal weight, 100 overweight women, 50 with Class 1 obesity and 30 with Class 2 obesity). Half of the women were pregnant before the COVID 19 pandemic, and their last menstruation occurred between1 January 2019 and 15 March 2019, whereas the other half became pregnant during the period of pandemic, and their last menstruation occurred between 1 January 2020 and 15 March 2020. Data on complications and the state of the newborn were obtained by doctors during a visit to the clinic during the puerperium.

The exclusion criteria constituted women in whom miscarriage occurred, intrauterine death, those suffering from chronic metabolic diseases, autoimmune neurological diseases, cardiovascular diseases, as well as having psychiatric treatment or being mentally retarded.

In accordance with Polish law [50] it is the duty of the physician providing care for a pregnant woman to make an assessment of the risk and intensity of depressive symptoms using the Beck depression inventory (BDI) twice in pregnancy, for the first time between week 11 and 14 of pregnancy, and in week 32 of pregnancy, in order to assess the risk of occurrence of postpartum depression [51].

Consent for the study was obtained from the Bioethical Committee at the Medical University in Lublin No. KE-0254/133/2019.

### 2.2. Assessment of Depression

The BDI is an instrument for assessment of the severity of symptoms of depression. The BDI is useful in diagnosing an episode of depression in accordance with the adopted diagnostic criteria, and monitoring emotional status. The questions concern the past 7 days, and include problems associated with emotional symptoms of depression, such as irritability, the feeling of hopelessness, and low self-esteem, as well as somatic symptoms, such as weight loss, insomnia, or fatigue. The Beck depression inventory, second edition is a self-report measure consisting of 21 items that are scored from 0 to 3. The severity of depression is determined by summing up the number of scores obtained. A higher score means a higher severity of depression, and is interpreted as follows: lack of depression (0–11 score), mild depression (12–19 score), moderate depression (20–25 score), and severe depression (26–63 score).

### 2.3. Statistical Methods

The data were analyzed using STATISTICA 13 software (Statsoft, Tulsa, OK, USA). The mean and standard deviation were estimated for numerical variables, as well as absolute numbers (n) and percentages (%) of the occurrence of items for categorical variables.

For the socio-economic characteristics of pre-pregnancy BMI groups, we used the Chi-square test to compare categorical characteristics between four groups of pre-pregnancy BMI, as well as the F test to compare numerical characteristics between four groups of pre-pregnancy BMI.

In analyses of the severity of depression, we used:

- Pearson’s correlation coefficient to correlate the severity of depression with BMI, severity of depression between the 10 and13th and 32nd gestational weeks, severity of depression with numerical socio-economic characteristics;
- Student’s paired *t* test to compare the severity of depression between the 10 and 13th and 32nd gestational weeks;
- two-factor analysis of variance with interaction to compare the severity of depression between four groups of pre-pregnancy BMI and the COVID-19 pandemic;
- Mann–Whitney’s U test to compare the severity of depression between before and during the COVID-19 pandemic in four groups of pre-pregnancy, separately;
- Student’s unpaired *t* test or F test analysis of variance to compare the severity of depression between categorical socio-economic characteristics, with two or more categories, respectively;

In analyses of the course of pregnancy and the state of newborns against pre-pregnancy BMI, the COVID-19 pandemic, and severity of depression, we used:

- regression models of gestational age at delivery or fetal weight against pre-pregnancy BMI, the COVID-19 pandemic, and severity of depression;
- logistic regression models of APGAR’s score (two categories (5–6 vs. 7–10) or pregnancy complication against pre-pregnancy BMI, COVID-19 pandemic, and severity of depression. In logistic regression models, we modeled one of the four complications during pregnancy versus none of these four complications.

We used both univariate and multivariate models. In univariate models we correlated one dependent variable with one independent variable (covariate), while in multivariate models we correlated one dependent variable with many independent variables (covariates). We used a forward step-by-step method to select covariates in regression models.

Due to the large sample size we assumed that the parameter estimators were asymptotically normally distributed due to the central limit theorem, so we used parametric tests.

The significance level was assumed to be 0.05 in all statistical tests.

## 3. Results

### 3.1. Characteristics of Pre-Pregnancy BMI Groups

Mean age of the examined women was 32.5 ± 5 years, the majority were urban inhabitants, were in a relationship, and had an average per capita household income. The above-mentioned characteristics did not significantly differ between pre-pregnancy BMI groups (Table 1). All women with normal body weight worked, followed by majority of those overweight (95%), with Class 1 obesity (84%), and only 40% of women with Class 2 obesity. The majority of women from all BMI groups performed intellectual work, women with normal weight and overweight until week 19 of pregnancy, while those who were obese stopped working sooner (those with Class 1 obesity until week 16, and those with Class 2 obesity until week 12).

### 3.2. Severity of Depression against Pre-Pregnancy BMI, COVID-19 Pandemic, and Socio-Economic Characteristics

Pre-pregnancy BMI was positively correlated with the severity of depression measured using the BDI, both in weeks 10–13 of pregnancy (*r* = 0.526, *p* < 0.001) and in week 32 of pregnancy (*r* = 0.539, *p* < 0.001). The higher the pre-pregnancy BMI, on average the higher the severity of depression during pregnancy.

The severity of depression in weeks 10–13 of pregnancy (12.74 ± 6.94) was significantly higher than in week 32 of pregnancy (15.02 ± 7.60, *p* < 0.001), and significantly positively correlated to each other (*r* = 0.973, *p* < 0.001).

The severity of depression both in weeks 10–13 and in week 32 of pregnancy was significantly different in the four groups of pre-pregnancy BMI, and correlated with COVID-19 pandemic. Moreover, the severity of depression was significantly dependent on the interaction between the four groups of pre-pregnancy BMI and COVID-19 pandemic (*p* = 0.048 for severity of depression in weeks 10–13 of pregnancy, and *p* = 0.011 in week 32).

**Figure 1:**
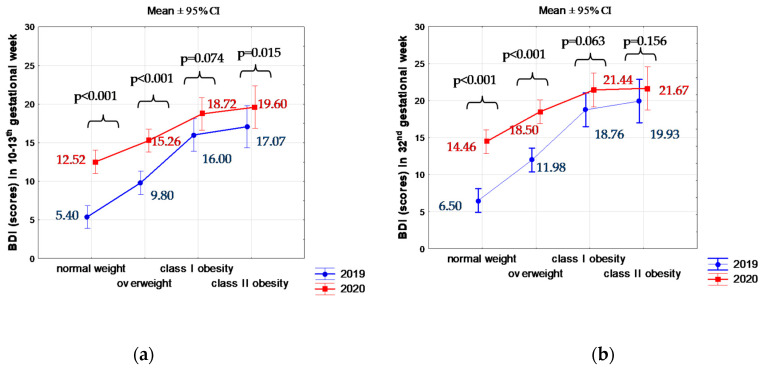
BDI in 10–13th (**a**) or 32nd (**b**) gestational weeks against pre-pregnancy BMI groups before and during COVID-19 pandemic. *p* for Mann–Whitney’s U test to compare BDI (scores) between before and during COVID-19 pandemic.

The severity of depression in weeks 10–13 of pregnancy was significantly higher during the pandemic than before pandemic (Figure 1a): in women with normal body weight before pregnancy (*p* < 0.001), those overweight (*p* < 0.001), and with Class 2 obesity (*p* = 0.015).

The severity of depression in week 32 of pregnancy was significantly higher during than before the pandemic (Figure 1b): in women with normal body weight before pregnancy (*p* < 0.001), and those overweight (*p* < 0.001).

The severity of depression in weeks 10–13 and 32 of pregnancy was positively correlated with age of the pregnant women (*r* = 0.404, *p* < 0.001 and *r* = 0.419, *p* < 0.001, respectively), but negatively correlated with duration of working during pregnancy (*r* = −0.258, *p* < 0.001 and *r* = −0.248, *p* < 0.001, respectively). The more severe the depression during pregnancy was, the older the pregnant women were, and the shorter they worked during pregnancy, on average.

More severe depression, both in weeks 10–13 and 32 of pregnancy, was noticed in the single pregnant women than in the pregnant women who were married or were in a relationship, as well as in non-working pregnant women than in working pregnant women.

However, the severity of depression in weeks 10–13 and 32 of pregnancy did not correlate with level of education, place of residence, and monthly income per capita in a household (Table 2).

### 3.3. Course of Pregnancy and State of a Newborn against Pre-Pregnancy BMI, COVID-19 Pandemic and Severity of Depression

Having analyzed covariates separately, we found that pre-pregnancy BMI and the severity of depression correlated negatively with gestational age at delivery and fetal weight (Table 3). The higher the pre-pregnancy BMI and the severity of depression were, the lower the gestational age at delivery and fetal weight, on average. Moreover, gestational age at delivery and fetal weight were significantly lower during the COVID-19 pandemic than before it.

In the multivariate analysis, we observed that only the severity of depression in the 10–13th gestational week was an independent factor that shortened gestational age at delivery. Pre-pregnancy BMI and the severity of depression in the 32nd gestational week were independent factors that reduced fetal weight.

Having analyzed covariates separately, we found that the odds of lower APGAR (5 or 6) were significantly higher during COVID-19 pandemic than before it. The odds of lower APGAR were in the women with higher pre-pregnancy BMI and higher severity of depression during pregnancy (Table 4).

In the multivariate analysis we observed that only the severity of depression in the 10–13th gestational week was an independent factor that increased the odds of lower APGAR.

Having analyzed covariates separately, we found that the odds of gestational diabetes, gestational protein uric hypertension, and intrauterine growth were significantly higher in the women with higher pre-pregnancy BMI and higher severity of depression during pregnancy, but not significantly different between during and before COVID-19 pandemic (Table 5).

The odds of gestational hypertension were significantly higher during COVID-19 pandemic than before it, and they were higher in the women with a higher severity of depression. The odds of gestational hypertension did not significantly correlate with pre-pregnancy BMI (Table 5).

In the multivariate analysis we observed that pre-pregnancy BMI and severity of depression in the 10–13th gestational week were independent factors that increased the risk of gestational diabetes. The severity of depression in the 10–13th gestational week was an independent factor that increased the risk of gestational hypertension and intrauterine growth, however the severity of depression in the 32nd gestational week was an independent factor that increased the risk of gestational protein uric hypertension (Table 6).

## 4. Discussion

Possible risk factors for the development of depression among mothers differ. Reports concerning possible changes in the body during pregnancy focused on the effect of depression on disorders within the hypothalamic–pituitary–adrenocortical axis (HPA), and secretion of cortisol and catecholamines which, in consequence, may lead to disorders in fetal growth and preterm delivery through disorders in the supply of nutrients to the fetus [52,53], as well as on the route, by increasing the concentration of corticotropin-releasing hormone (CRH), which participates in maternal–fetal neuroendocrine, immune, and metabolic responses, and which, in consequence, may result in pre term birth (PTB) [54].

In the presented study, both among overweight and obese women, the symptoms of depression considerably intensified between the measurements in weeks 10–13 of pregnancy and week 32 of pregnancy. An increase in intensity of depression with the duration of pregnancy among obese women was also reported by Kumpulainen et al. [8], Salehi-Pourmehr et al. [10], and Bogaerts et al. [55].

An increase in depressive symptoms also affected women in whom, during pregnancy, there occurred complications, such as GDM (gestational diabetes), gestational HT (hypertension), gestational hypertension with proteinuria, and fetal IUGR (intrauterine growth restriction) during both measurements, compared to women without complications. This confirms the theory of co-occurrence of depression with various pathological conditions during pregnancy [56,57]. Intensification of depressive symptoms in the case of an occurrence of pregnancy complications is equivocally defined in the literature. In a study by Kumpulainen et al., the result concerning an intensity of depression according to BMI was insignificant [8]. However, it was also insignificant in women with normal weight, whereas other researchers indicated the presence of a relationship and the possibility of an intensification of symptoms [58,59]. With respect to the results of the presented study we are inclined towards connecting pregnancy complications and the intensity of depressive symptoms.

First reports pertaining to the psychological status of pregnant women during the pandemic have indicated an increase in the frequency of occurrence of depression during pregnancy, and the results of the presented study are in accordance with these reports by Ayaz et al. [43], Lopez-Morales et al. [60], Rahimi et al. [61], and Hessami et al. [62]. Women qualified for the study during the COVID-19 pandemic were characterized by a significantly higher level of depressive symptoms than those examined before the pandemic. This relationship concerned women in each BMI interval during the first measurement in the first trimester of pregnancy. In the third trimester of pregnancy an increase in depression was observed in the group of women with normal weight and those overweight, whereas among women with Class 1 and Class 2 obesity the change was insignificant; however, the percentage of pregnant women with depressive symptoms was anyway significantly higher than in the remaining groups. The authors share the opinion that increased feelings of fear and anxiety among pregnant women caused by worries about the state of health of themselves and their babies as an adverse effect of COVID-19 infection during pregnancy have not yet been well described. In addition, the necessity for greater social isolation may have exerted a negative effect on the psychological state of the mothers, increasing depressive symptoms, generalized anxiety disorder, insomnia, and stress [63,64].

The researchers obtained access to two studies investigating the problem of co-occurrence of depression and obesity and their effect on neonatal outcomes [65,66]. However, considering the relative scarceness of reports concerning the psychological status of pregnant women during the pandemic this study seems to be the first attempt at investigating the effect of both depressive symptoms and obesity on neonatal outcomes with relation to COVID-19.

The presented study confirmed the effect of excessive body weight before pregnancy on the risk of occurrence of gestational diabetes. This is in accordance with observations described by Pinheiro et al. [67], Huet et al. [68], and Farren et al. [69]. The pathomechanism of the observed phenomenon may be the reduction of the reduction of adiponectin level in visceral obesity, which has a diabetogenic and atherogenic effect, because adiponectin increases insulin sensitivity, and has an anti-inflammatory effect [70].

The presented study also demonstrated that excessive body weight favors the occurrence of gestational diabetes, as well as gestational protein uric hypertension. The connection between these two pregnancy complications was confirmed by the review by Weissgerber et al., who found that preeclampsia [71] risk is increased two to four-fold among women with type 1 or type 2 diabetes. It was observed that GDM is also associated with an increased risk of preeclampsia; however, it is uncertain if the pathophysiological pathway of these two conditions is the same. In addition, women without diabetes who had developed preeclampsia were more predisposed to diabetes type 2 in future. Despite the fact that the mechanisms through which obesity increases the risk of preeclampsia have not been fully explained, some data strongly suggest that those involved concern: activated macrophages and uterine natural killer cells, which perform a crucial role in placental development; the activation of peripheral T helper cells inducing the production of cytokines, including TNF-α, IL-6 and IL-17; as well as the anti-angiogenic agent sFlt-1, and B cells that produce angiotensin type 1 receptor agonistic autoantibodies (AT1-aa).

The results obtained suggest that an excessive body weight before pregnancy is conducive to an earlier termination of pregnancy. Preterm birth is the leading cause of death and is, to a great extent, responsible for long-term loss of human potential among survivors throughout the world. Relationships similar to those obtained in the presented study, concerning an unfavorable effect of BMI on preterm delivery, were reported by Liu B. et al. [72], who analyzed 7,141,630 cases from the US National Vital Statistics System for 2016 and 2017; however, the risk differed according to maternal age, and race or ethnicity. In our study the women did not differ ethnically. Similar observations were reported by Slack et al. [73], Kurz et al. [74], and Kanadys et al. [75]. In the presented study weight gain in pregnancy was not analyzed from the perspective of preterm labor, nevertheless, in a study by Goldstein RF et al., gestational weight gain greater than, or less than, guideline recommendations, compared with weight gain within the recommended levels, was associated with higher risk of adverse maternal and infant outcomes [76]. Tersigni et al. [77] suggested that tissue inflammation might represent the pathogenic link between maternal obesity and increased occurrence of preterm delivery. This was confirmed in a study conducted by Lin Y. et al. carried out on animal models [78].

The presented study demonstrated that an excessive body weight of the mother before pregnancy results in the occurrence of high birth weight of a baby, which was confirmed by many reports presented in a review by Catalano et al. [79]. A study by Metzger et al. [80] analyzed data from more than 23,000 women in the Hyperglycemia and Adverse Pregnancy Outcomes (HAPO) study, and found that the prevalence of macrosomia among 17,244 non-obese women without GDM was 6.7% compared to 10.2% in 2791 non-obese women with GDM, and 20.2% in 935 obese women with GDM. Women who did not have GDM but who were obese had a 13.6% increased risk of macrosomia (defined as a child weighing 4000 g or more at birth) than non-obese women [80]. High birth weight of the baby may be explained by the fact that starting from the second trimester, the pancreas of the baby secretes insulin in response to hyperglycemia, which results in hyperinsulinemia. Combined, hyperglycemia and hyperinsulinemia account for an increase in the fat and protein stores of the fetus, and result in macrosomia.

Based on the results of our study it was found that an excessive body weight of the mother before delivery exerts a negative effect on the state of the newborn, measured by the APGAR scale. We found a significant negative correlation between APGAR and pre-pregnancy BMI (*r* = −0.262, *p* < 0.001). This is in accordance with the results of the systematic review and meta-analysis by Zhu T. E et al. [81].

The results of the pooled analysis performed by the researchers showed that the following factors are associated with an Apgar score <7 at the 5th minute: overweight (OR 1.13; 95% CI, 1.08–1.20), obese (OR 1.40; 95% CI, 1.27–1.54), and very obese (OR 1.71; 95% CI, 1.55–1.89) It was also found that overweightness or obesity of the mother increased the risk for Apgar score <7 at the 1st minute. A strong relationship was noted between a mother’s BMI in early pregnancy and fat mass, including visceral fat mass. There are many pathways by which obesity of the mother may exert an effect on the state of the newborn immediately after birth. The placenta is susceptible to lipid accretion related with obesity [82]. In obese women the placental lipid accumulation is 50% higher compared to slim women [83], which increases the risk of placental insufficiency. Furthermore, lipotoxicity may be involved in the pathogenesis of the placenta through placental oxidative stress and inflammatory response [84].

In the presented study intensification of depressive symptoms considerably shortened the duration of pregnancy. Many researchers have indicated that depression in pregnancy exerts an effect on the risk of preterm delivery or reduces gestational age at delivery [17,59,85]. Other studies have shown that in the European environment and with good family income this effect is insignificant [85]. In our study this relationship, for Polish women, proved to be true.

Depression in pregnancy is considered as one of the risk factors for low birth weight babies [86]. The results of the presented study confirmed a reduction in fetal weight with intensification of depression symptoms, which is in accordance with studies by Alder et al. [17], Grote et al. [85], and Ogunyemi et al. [59].

However, no effect of depression was observed among the examined women with IUGR, despite the fact that in women diagnosed with IUGR depressive symptoms intensified. Grote et al., in a meta-analysis of studies concerning the effect of depression in pregnancy on neonatal outcomes, did not confirm the relationship between depression and IUGR, and this relationship was also not confirmed by other studies [17,59,85]. Although in recent meta-analyses there have been reports concerning the effect of depression on IUGR [87], these studies did not consider the division of obese women with depression. Apart from an earlier date of delivery, the reduction of the weight of the baby during pregnancy may be due to the improper state of nutrition associated with the above-mentioned increase in CRH secretion.

In the presented study, babies of mothers with depression obtained lower APGAR scores than babies of mothers on the threshold of the recommended weight. The same observations were reported by McDonald et al. [66], who examined 70,605 women and found that the differences in lower APGAR score were especially clear among obese women with depressive symptoms [59,65]. Probably this can be explained by the negative effect of decreased oxytocin secretion via the hypothalamic–pituitary–adrenocortical axis, and by increased noradrenaline secretion, which may cause hypoxia in a newborn during delivery [32].

A limitation of our study is that the covariates of mental health in obese women were not considered, which may exert an effect on depression rates together with obesity, e.g., insufficient sleep, shift work, low self-efficacy, or low social support [8]. There are many other factors that could affect depression. Another possible limitation of this study is a lack of correlation between depression and other socio-economic data and data about perinatal diseases that appeared during pregnancy. These data, and the type and duration of delivery might increase the symptoms of depression and may effect a newborn.

Summing up, depression and obesity in pregnancy have a negative effect on its course. Obese women are characterized by increased symptoms of depression. In addition, obesity results in the occurrence of pregnancy complications, which enhance depressive symptoms in pregnant women. Before the COVID-19 pandemic the intensity of depressive symptoms among the examined women was lower than after its onset. Thus, during this period special attention should be devoted to women with overweightness and obesity, because in these women intense depressive symptoms in pregnancy exert a negative effect on the duration of pregnancy, growth of the baby, and APGAR scores. Considering an excessive increase in maternal and fetal concomitant diseases and pregnancy complications during the pandemic, the quality of perinatal care should be adjusted to the existing situation through increasing pregnant women’s access to medical care. However, this raises the need for confirmation of these observations in further studies including a larger number of cases.

## 5. Conclusions

Excessive body weight before pregnancy is conducive to depressive disorders during pregnancy. Depressive symptoms among pregnant women with overweightness and obesity intensified during the COVID-19 pandemic. Elevated BMI while becoming pregnant, and during pregnancy increases the risk of gestational diabetes, gestational protein uric hypertension, preterm delivery, and has a negative effect on the state of a newborn measured by APGAR scale. Antenatal depression increases the risk of preterm delivery, and exerts a negative effect on the state of a newborn measured by APGAR scale. The COVID 19 pandemic is a factor which increases the severity of depressive symptoms in pregnant women.

## Figures and Tables

**Table 1:** Socio-economic characteristics of pre-pregnancy BMI groups.

Variable, Parameter		Normal Weight (*n* = 100)	Overweight (*n* = 100)	Class I Obesity (*n* = 50)	Class II Obesity (*n* = 30)	*p*
Age (years), M ± SD		32.49 ± 4.98	32.64 ± 4.90	32.76 ± 5.12	32.63 ± 5.29	0.991
Level of education, *n* (%)	primary	11 (11.00)	10 (10.00)	5 (10.00)	2 (6.67)	0.988
	secondary	59 (59.00)	56 (56.00)	29 (58.00)	17 (56.67)	
	tertiary	30 (30.00)	34 (34.00)	16 (32.00)	11 (36.67)	
Place of residence, *n* (%)	village	30 (30.00)	25 (25.00)	14 (28.00)	7 (23.33)	0.763
	town	33 (33.00)	41 (41.00)	22 (44.00)	14 (46.67)	
	city	37 (37.00)	34 (34.00)	14 (28.00)	9 (30.00)	
Marital status, *n* (%)	single	8 (8.00)	11 (11.00)	8 (16.00)	5 (16.67)	0.388
	married or in relationship	92 (92.00)	89 (89.00)	42 (84.00)	25 (83.33)	
Monthly income per capita in household (thousands PLN), *n* (%)	below 2	11 (11.00)	18 (18.00)	10 (20.00)	10 (33.33)	0.133
	2–3.5	59 (59.00)	51 (51.00)	24 (48.00)	15 (50.00)	
	over 3.5	30 (30.00)	31 (31.00)	16 (32.00)	5 (16.67)	
Working, *n* (%)		100 (100.00)	95 (95.00)	42 (84.00)	12 (40.00)	<0.001
Type of job, *n* (%) *	physical	25 (25.00)	22 (23.16)	17 (40.48)	6 (50.00)	0.060
	intellectual	66 (66.00)	70 (73.68)	24 (57.14)	6 (50.00)	
	mixed	9 (9.00)	3 (3.16)	1 (2.38)	0 (0.00)	
Working until week, M ± SD *		18.94 ± 10.81	19.52 ± 10.83	16.64 ± 10.27	11.92 ± 3.87	0.072

M—mean, SD—standard deviation, BMI—body mass index, *p* for Chi-square test for categorical variables or F test for numerical variables, * calculated for working women only.

**Table 2:** Severity of depression against socio-economic characteristics.

Variable	IU or Category	Parameter	BDI (Scores)			
			in 10–13th Gestational Week		in 32nd Gestational Week	
			Result of Parameter	*p*	Result of Parameter	*p*
Age	years	*r*	0.404	<0.001	0.419	<0.001
Level of education	primary	M ± SD	12.36 ± 7.27	0.913	14.89 ± 8.01	0.996
	secondary	M ± SD	12.68 ± 6.86		15.01 ± 7.49	
	tertiary	M ± SD	12.96 ± 7.04		15.03 ± 7.74	
Place of residence	village	M ± SD	11.91 ± 7.46	0.252	13.97 ± 7.98	0.210
	town	M ± SD	13.55 ± 7.05		15.93 ± 7.78	
	city	M ± SD	12.46 ± 6.31		14.76 ± 7.00	
Marital status	single	M ± SD	18.78 ± 4.65	<0.001	22.09 ± 4.84	<0.001
	married or in relationship	M ± SD	11.96 ± 6.81		14.06 ± 7.41	
Monthly income per capita in household	thousands PLN	*r*	−0.067	0.187	−0.081	0.138
Working	yes	M ± SD	12.11 ± 6.98	<0.001	14.33 ± 7.67	<0.001
	no	M ± SD	17.81 ± 3.85		20.45 ± 3.93	
Working until week *		r	−0.258	<0.001	−0.248	<0.001

M—mean, SD—standard deviation, BDI—Beck depression inventory, *r*—Pearson’s correlation coefficient, F test analysis of variance for level of education or place of residence, Student’s *t* test for marital status or working. * calculated for working women only.

**Table 3:** Univariate and multivariate regression models of gestational age at delivery or fetal weight against pre-pregnancy BMI, COVID-19 pandemic, and severity of depression.

Covariate	Gestational Age at Delivery (Weeks)				Fetal Weight (g)			
	Univariate Models		Multivariate Model		Univariate Models		Multivariate Model	
	b	*p*	b	*p*	b	*p*	b	*p*
Pre-pregnancy BMI (kg/m^2^)	−0.07	0.008	-	ns.	−40.56	<0.001	−15.57	0.042
COVID-19 pandemic (yes vs. no)	−0.87	0.001	-	ns.	−184.72	0.010	-	ns.
BDI (scores) in 10–13th gestational week	−0.12	<0.001	−0.12	<0.001	−39.10	<0.001	-	ns.
BDI (scores) in 32nd gestational week	−0.11	<0.001	-	ns.	−36.07	<0.001	−30.55	<0.001

BMI—body mass index, BDI—Beck depression inventory, b—mean change in dependent variable per unit of covariate, ns—not significant.

**Table 4:** Univariate and multivariate logistic regression models of APGAR’s score against pre-pregnancy BMI, COVID-19 pandemic, and severity of depression.

Covariate	APGAR 5–6 (*n* = 65) vs. 7–10 (*n* = 215)			
	Univariate Models		Multivariate Model	
	OR	*p*	OR	*p*
Pre-pregnancy BMI (kg/m^2^)	1.0765	0.009	-	ns.
COVID-19 pandemic (yes vs. no)	3.1765	0.009	-	ns.
BDI (scores) in 10–13th gestational week	1.1729	<0.001	1.1729	<0.001
BDI (scores) in 32nd gestational week	1.1554	<0.001	-	ns.

BMI—body mass index, BDI—Beck depression inventory, OR—odds ratio, ns—not significant.

**Table 5:** Univariate logistic regression models of pregnancy complication against pre-pregnancy BMI, COVID-19 pandemic, and severity of depression.

Covariate	Gestational Diabetes (*n* = 51)		Gestational Hypertension (*n* = 14)		Gestational Protein uric Hypertension (*n* = 24)		Intrauterine Growth (*n* = 30)	
	vs. no Complication during Pregnancy (*n* = 195)							
	OR	*p*	OR	*p*	OR	*p*	OR	*p*
Pre-pregnancy BMI (kg/m^2^)	1.2136	<0.001	1.0869	0.153	1.2027	<0.001	1.1615	<0.001
COVID-19 pandemic (yes vs. no)	1.0398	0.877	3.6729	0.028	1.1819	0.999	1.5006	0.246
BDI (scores) in 10–13th gestational week	1.1534	<0.001	1.3320	<0.001	1.1720	<0.001	1.1918	<0.001
BDI (scores) in 32nd gestational week	1.1334	<0.001	1.2623	<0.001	1.1714	<0.001	1.1654	<0.001

BMI—body mass index, BDI—Beck depression inventory, OR—odds ratio.

**Table 6:** Multivariate logistic regression models of pregnancy complication against pre-pregnancy BMI, COVID-19 pandemic, and severity of depression.

Covariate	Gestational Diabetes (*n* = 51)		Gestational Hypertension (*n* = 14)		Gestational Protein uric Hypertension (*n* = 24)		Intrauterine Growth (*n* = 30)	
	vs. no Complication during Pregnancy (*n* = 195)							
	OR	*p*	OR	*p*	OR	*p*	OR	*p*
Pre-pregnancy BMI (kg/m^2^)	1.1436	0.002	-	ns.	-	ns.	-	ns.
COVID-19 pandemic (yes vs. no)	-	ns.	-	ns.	-	ns.	-	ns.
BDI (scores) in 10–13th gestational week	1.1046	0.002	1.3316	<0.001	-	ns.	1.1918	<0.001
BDI (scores) in 32nd gestational week	-	ns.	-	ns.	1.1714	<0.001	-	ns.

BMI—body mass index, BDI—Beck depression inventory, OR—odds ratio, ns—not significant.

## Data Availability

The data presented in this study are available on request from Wdowiak Artur. The data are not publicly available due to privacy restrictions.
